# Accelerated Idioventricular Rhythm: History and Chronology of the Main Discoveries

**Published:** 2010-01-07

**Authors:** Andres Ricardo Perez Riera, Raimundo Barbosa Barros, Francisco Daniel de Sousa, Adrian Baranchuk

**Affiliations:** 1 ABC Medical Faculty. ABC Foundation. Santo Andre - Sao Paulo. Brazil; 2 Hospital de Messejana Dr. Carlos Alberto Studart Gomes, Fortaleza-Ceara-Brazil; 3 Kingston General Hospital, Queen's University, Kingston, Ontario, Canada

**Keywords:** Accelerated Idioventricular Rhythm, History and Chronology, Slow ventricular tachycardia

## Abstract

Accelerated Idioventricular Rhythm (AIVR) is a ventricular rhythm consisting of three or more consecutive monomorphic beats, with gradual onset and gradual termination. It can rarely manifest in patients with completely normal hearts or with structural heart disease. It is usually seen during acute myocardial infarction reperfusion. This manuscript aims to review the history of the main discoveries that lead to the identification and comprehension of this fascinating arrhythmia.

##  Introduction - Definitions

The so called "Accelerated Idioventricular Rhythm" (AIVR) is a ventricular rhythm (originating from the His, the Purkinje system or the working contractile ventricular cells) consisting of three or more consecutive monomorphic beats, with gradual onset. Less commonly, AIVR is polymorphic. The discharge rate of the ectopic focus is similar to the sinus rate (isorhythmic) between 50 and 120 bpm. The ectopic focus manifests when the sinus rate slows down (below the ectopic focus) or when the ectopic focus accelerates above the intrinsic rate by 30-40 beats per minute. When both discharge rates (sinus and ectopic focus) are similar, isorhythmic dissociation, fusion and capture beats can be seen.

AIVR is usually a benign and well-tolerated arrhythmia. Most of the cases will require no treatment and in rare situations such as sustained or incessant AIVR or when AV dissociation induces syncope, the risk of sudden death is higher, and the arrhythmia should be treated.

Different terminology was used to describe AIVR: Non paroxysmal ventricular tachycardia (VT), isorhythmic slow VT, and the curious benevolent tachycardia.

Non paroxysmal VT is currently a non accepted definition given the fact that AIVR is usually a ventricular rhythm at rate slower than 100 bpm, thus not meeting criteria to be called tachycardia.

## AIVR - Clinical Scenarios

Rarely, AIVR can be detected in the youth and, in this subgroup, is usually benign [1] and requires no specific treatment.

AIVR occurs infrequently in patients without demonstrable heart disease and, in this case, it has a good prognosis. When AIVR presents in individuals with no structural heart disease, the mechanism involved is usually an increased vagal tone and decreased sympathetic tone [2], as observed in athletes. The acceleration of an independent focus during exercise strongly suggests that this ectopic focus is under autonomic influence, a phenomenon also seen during central nervous system (CNS) stimulation in dogs [3]. A similar mechanism has also been described for pregnant women in the antenatal period [4].

AIVR is frequently observed during the reperfusion phase that follows an acute myocardial infarction (AMI) [5]. It remains controversial whether or not, AIVR implies complete reperfusion of the culprit lesion or only some degree of reperfusion.  AIVR has been also described associated with several drug intoxications such as halothane [6], aconitine [7], desflurane [8], cocaine [9], and digitalis [10-12]. Electrolyte imbalance has also been associated with AIVR in an experimental study [13]. There are also case reports published on the association of AIVR with the post-resuscitation period [14] (as an initial resolving rhythm), in chronic ischemic and non ischemic dilated cardiomyopathy [15], and less frequently in cardiomyopathies such as hypertrophic cardiomyopathy [16],  arrhythmogenic right ventricular dysplasia (ARVD) [17], and in newborn infants with different congenital heart diseases [18].

**Figure 1:**
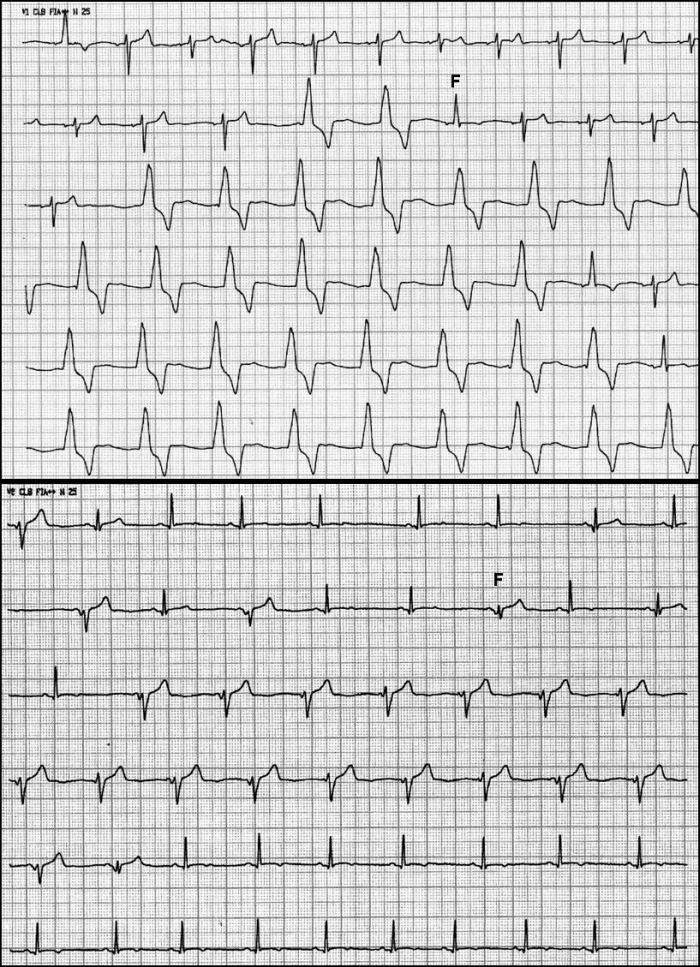
This ECG belongs to a 23 year-old male patient, Caucasian, asymptomatic.  No cardiovascular history. He practiced physical activities regularly. He has been treated with azitromicine for an upper airway infection. Both panels show sinus rhythm alternating with AIVR at approximately 60 bpm. Note fusion beats (F).

**Figure 2:**
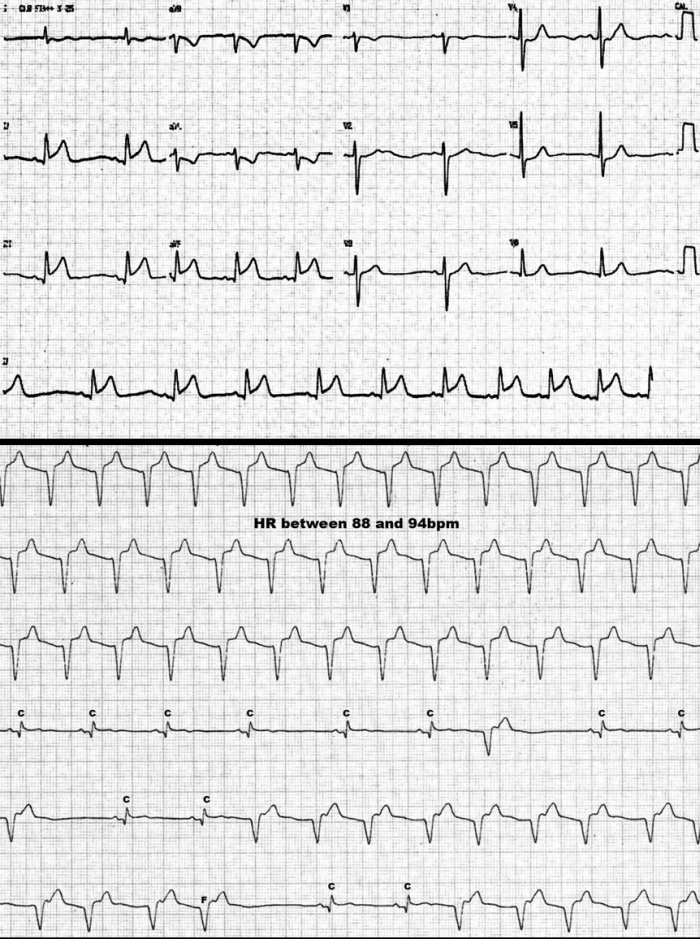
This ECG belongs to a 67 year-old woman, presenting to the ER with an acute inferior MI (superior panel). During the administration of thrombolytics, AIVR can be seen (inferior panel) at rate of 88 bpm. AIVR alternates with sinus captures (C) and fusion beats (F).

A typical example of AIVR in the setting of normal structural heart can be seen in Figure 1. In Figure 2, a case of AIVR in the context of a reperfused acute inferior MI can be seen.

## Mechanisms

The main electrophysiological mechanism involved in AIVR is an abnormal calcium-dependent automatism (ectopic automaticity) that affect phase 4 of action potential (diastolic depolarization) [19]. When AIVR is associated with digitalis intoxication, the main arrhythmogenic mechanism involved is trigger activity [20].

This manuscript aims to be a chronological historical review on the main findings that contributed to the understanding of this fascinating arrhythmia.

## Chronology of discoveries

### 1925:

Sir Thomas Lewis (December 26th, 1881, Cardiff, Gales - March 17th 1945) a pioneer of the British cardiology showed the first ECG trace of AIVR case in his book entitled "Mechanism and graphic registration of the heartbeat"  [21], dedicated to Willem Einthoven. He failed to identify AIVR as an independent arrhythmia. This book is considered the first book on Electrocardiography, as highlighted by Prof. Schapiro [22] a well-recognized historian in electrocardiology and arrhythmias. Several years prior to the publication of his book, Sir Lewis reported in The Lancet (1909) an interesting article about the mechanisms of VT without mentioning its slower forms [23].

### 1950:

Harris [24] was the first one to identify AIVR associated with ischemia and reperfusion in an experimental study. He observed this rhythm after clamping the right coronary artery. He identified the origin of this rhythm in the ventricles and described a similar heart rate than the sinus discharge. He also mentioned that this rhythm may overrate the sinus rate but also could be inhibited by increasing the sinus discharge.

### 1966:

Mariot and Menendez [25] have introduced the currently accepted terminology: AIVR.

### 1967:

Dessertene [26] described for the first time the term "slow ventricular tachycardia"

### 1969:

Agustín Castellanos Jr [19] used the term "slow ventricular tachycardia" during an acute MI to describe a case of AIVR. Additionally, the authors explained the underlying electrophysiological mechanisms of AIVR.

### 1973:

Rothfeld et al [27] showed for the first time the coexistence of paroxysmal ventricular tachycardia and "idioventricular rhythm" during an acute MI.

### 1974:

Rothfeld and Zucker [28], were the first ones to describe the polymorphic form of AIVR. They named it "Multiform accelerated idioventricular rhythm". Sclarowski, 4 years later (1978), published 2 cases with polymorphic presentation in the Journal of Electrocardiology [29].

### 1975:

Agustín Castellanos Jr et al [10] reported digitalis-induced AIVR for the first time in the literature.  Scheinman et al [30] reported the efficacy of atropine to suppress AIVR during the acute MI. Basy and Scheinman [31] reported the sustained form of AIVR in an elderly patient suffering an inferior MI. The authors successfully treated the patient with IV Procainamide. The same year, Castellanos et al [32] reported an AIVR from two different foci. They named it "double ectopic accelerated ventricular rhythm".

### 1976:

Hasin and Rogel [33] presented the currently accepted mechanism involved in AIVR. They postulated the involvement of the phase 4 slope of the action potential (ectopic automaticity) for the unstable form of AIVR that is frequently observed in  acute MI. The same year, Doshchitsin and Merkulova from Russia [34], used the terminology "non-paroxysmal ventricular tachycardia".

### 1980:

Delise et al [15] reported the first case of AIVR associated with hypertrophic cardiomyopathy.

### 1981:

Bernard et al [1], described AIVR in a pediatric patient. They highlighted the usual benign course of this arrhythmia in this population.

### 1983:

Sclarovsky et al [35], showed a partial benefit of eliminating AIVR by treating a patient with Verapamil. The same year, Golderberg et al [36], reported that the presence of AIVR during the acute phase of a MI treated with thrombolytic therapy, rather than a malignant sign, implies reperfusion of the culprit lesion. The sensitivity is low but the specificity is high (> 80%). Jonsson et al [9] described the association of AIVR with the use of cocaine.

### 1987:

Chiale et al [37], reported a case of slow automaticity in a young patient with no structural heart disease. 

### 1988:

Martini et al [16] described the first case of AIVR originating in the infundibular region of the right ventricle in a patient with a concealed form of arrhythmogenic right ventricular dysplasia (ARVD).

### 1991:

Tatu-Chitoiu [38], used the term "Non-paroxysmal ventricular tachycardia with isorhythmic atrioventricular dissociation" to described AIVR.

### 1993:

Nakawa et al [18], showed that AIVR had no impact in long-term prognosis in a large population of pediatric patients with congenital heart disease. The same year, Martinez-Lopez JI et al [39], created the term "benevolent rhythm" to describe AIVR. This term has not convinced the cardiology community and currently is not been used.

### 2000:

Grimm et al [14] followed up a series of patients with AIVR with idiopathic dilated cardiomyopathy. The presence of the arrhythmia did not impact on long-term prognosis.

### 2002:

Marret et al [8] reported a case of AIVR associated with desflurane intoxication.

### 2004:

Dulac et al [4], reported the first case of AIVR in a pregnant woman in the antenatal period.

### 2005:

Bonnemeier et al [2], determined that AIVR is not a specific marker of reperfusion during an acute MI treated with thrombolytics. The presence of AIVR could not distinguish between complete or incomplete reperfusion. They established a connection between AIVR and autonomic nervous system imbalance, AIVR being more frequently associated with increased parasympathetic tone and decreased sympathetic tone. The authors confirmed that the presence of AIVR did not affect the short or long-term prognosis.

### 2007:

Tsai et al [13] reported the first case of AIVR in a post-resuscitated patient.  The same year, Nasir et al [40], reported cases of AIVR in elite athletes. Again, the authors related the presence of the arrhythmia with autonomic dysfunction (hypervagotonia). Fuyita et al [7] reported a case of AIVR associated with intoxication with acotinine.

### 2008:

Hsu et al [41], reported the first case of AIVR associated with Buerger disease (also known as thromboangiitis obliterans) and MI. In the same year, Chhabra et al [6] reported a case of AIVR observed during inhalational induction with halothane in a child with congenital cataract. Osmancik et al  [42], confirmed that the value of AIVR alone as a criteria for reperfusion is low, but in addition to normalization of the ST-segment, the probability of successful reperfusion is higher.

## Conclusions

This intriguing arrhythmia could manifest in several different clinical scenarios. There are benign forms in subjects with no structural heart disease in which case, it usually requires no intervention, but sometimes it can present as a more severe arrhythmia, requiring treatment. Since its initial identification by Harris, major advances have been done in the understanding on the underlying mechanisms involved in the genesis of this arrhythmia. Abnormal calcium-dependent automatism that affects the phase 4 slope of the action potential (ectopic automaticity) has been identified as the main electrophysiological mechanism involved in AIVR. Autonomic imbalance has also been identified as a trigger of this abnormal rhythm. More recent reports showed that the presence of AIVR does not constitute a specific marker of complete reperfusion during the acute phase of a myocardial infarction, but it could indicate that the responsible vessel is open.  In this context, AIVR usually does not require specific treatment.
